# PEG grafted chitosan scaffold for dual growth factor delivery for enhanced wound healing

**DOI:** 10.1038/s41598-019-55214-7

**Published:** 2019-12-16

**Authors:** Amritha Vijayan, Sabareeswaran A., G. S. Vinod Kumar

**Affiliations:** 1 Cancer Biology, Nano Drug Delivery Systems (NDDS), Bio-Innovation Center (BIC), Rajiv Gandhi Centre for Biotechnology, Thycaud P.O, Thiruvananthapuram, Kerala 695014 India; 2 Histopathology laboratory, Sree Chitra Tirunal Institute for Medical Sciences & Technology, Thiruvananthapuram, Kerala 695011 India; 3 Research Scholar, Department of Biotechnology, Faculty of Applied Science & Technology, University of Kerala, Trivandrum, Kerala 695581 India

**Keywords:** Biomaterials, Translational research

## Abstract

Application of growth factors at wound site has improved the efficiency and quality of healing. Basic fibroblast growth factor (bFGF) and vascular endothelial growth factor (VEGF) induce proliferation of various cells in wound healing. Delivery of growth factor from controlled release systems protect it from degradation and also result in sustained delivery of it at the site of injury. The goal of the study was to develop a Polyethylene glycol (PEG) cross-linked cotton-like chitosan scaffold (CS-PEG-H) by freeze-drying method and chemically conjugate heparin to the scaffold to which the growth factors can be electrostatically bound and evaluate its wound healing properties *in vitro* and *in vivo*. The growth factor containing scaffolds induced increased proliferation of HaCaT cells, increased neovascularization and collagen formation seen by H and E and Masson’s trichrome staining. Immunohistochemistry was performed using the Ki67 marker which increased proliferation of cells in growth factor containing scaffold treated group. Frequent dressing changes are a major deterrent to proper wound healing. Our system was found to release both VEGF and bFGF in a continuous manner and attained stability after 7 days. Thus our system can maintain therapeutic levels of growth factor at the wound bed thereby avoiding the need for daily applications and frequent dressing changes. Thus, it can be a promising candidate for wound healing.

## Introduction

Wound healing is a complex process which mainly consists of three phases like inflammation, proliferation and tissue remodeling. Inflammatory cells and stromal cells secrete growth factors at the site of injury which helps in all phases of wound healing^1^. Application of growth factors at the site of the wound has shown to improve the efficiency and quality of wound healing as they stimulate angiogenesis and proliferation of cells and that in turn regulates the production and degradation of the extracellular matrix^2,3^. Local application of growth factors has been found to have poor efficiency due to their short half-life and rapid dilution in the body. They are also quickly degraded and inactivated by various factors at the site of injury. Growth factors have also proved to have undesirable side effects at high systemic levels^4^. Delivery of growth factor from controlled release systems has been found to overcome these problems as they protect the growth factors from degradation and also result in sustained delivery of growth factors at the site of injury.

Chitosan has been used in the treatment of wounds because of its anti microbial, hemostatic and muco adhersive properties^5–9^. It is said to accelerate fibroblast formation and help in the early phases of healing^10^. Chitosan has been shown to promote tissue growth and differentiation during wound healing, but its use in wound management is limited because of its poor mechanical properties^11–13^. Adding synthetic polymers like Polyethylene glycol (PEG) results in improved strength and elasticity^14–16^.

Vascular endothelial growth factor (VEGF) and basic fibroblast growth factor (bFGF) have been shown to induce proliferation of endothelial cells, fibroblasts and keratinocytes^17,18^. Clinical trials on wound healing of ulcers by topical administration of growth factors including bFGF and VEGF have largely been unsuccessful due to their short half-life, and at high systemic levels, they have been found to have undesirable effects^19^.

Adsorption of growth factors to the scaffold can be done by intermediate molecules like heparin which can be chemically conjugated to the scaffold^20^. Heparin is expressed abundantly at the site of injury. It helps wound healing process by preventing coagulation and activating various pro- and anti-inflammatory pathways. It also binds to growth factors such as VEGF and bFGF that stimulate the proliferation of various cells associated with wound healing^21,22^. Heparin binds to growth factors having heparin binding domains with high affinity by electrostatic interaction between the arginine and lysine residues of the growth factors and the negatively charged N- and O-sulfated groups of heparin^23,24^. These extend the half-lives of the growth factors by protecting them from proteolytic degradation and also increase their bioactivity^25–28^. Incorporation of growth factors using heparin which was covalently bound to a porous scaffold showed multifold improvement in vascularization^29^. Heparin is immobilized throughout the scaffold because of which there is a uniform distribution of growth factor in the scaffold.

To overcome the poor mechanical properties of chitosan, PEG was conjugated to chitosan in the presence of formaldehyde. Heparin was bound to the residual amine groups of chitosan by 1-Ethyl-3-(3-dimethylaminopropyl) carbodiimide (EDC) chemistry to form CS-PEG-H scaffold. Basic fibroblast growth factor (bFGF) and vascular endothelial growth factor (VEGF) were bound to the CS-PEG-H scaffold by the strong electrostatic interactions between growth factors and the negatively charged heparin. The wound healing properties of the scaffold were then evaluated by *in vitro* and *in vivo* methods.

## Results

### Biomaterial synthesis and characterization

#### IR spectra

**Figure 1:**
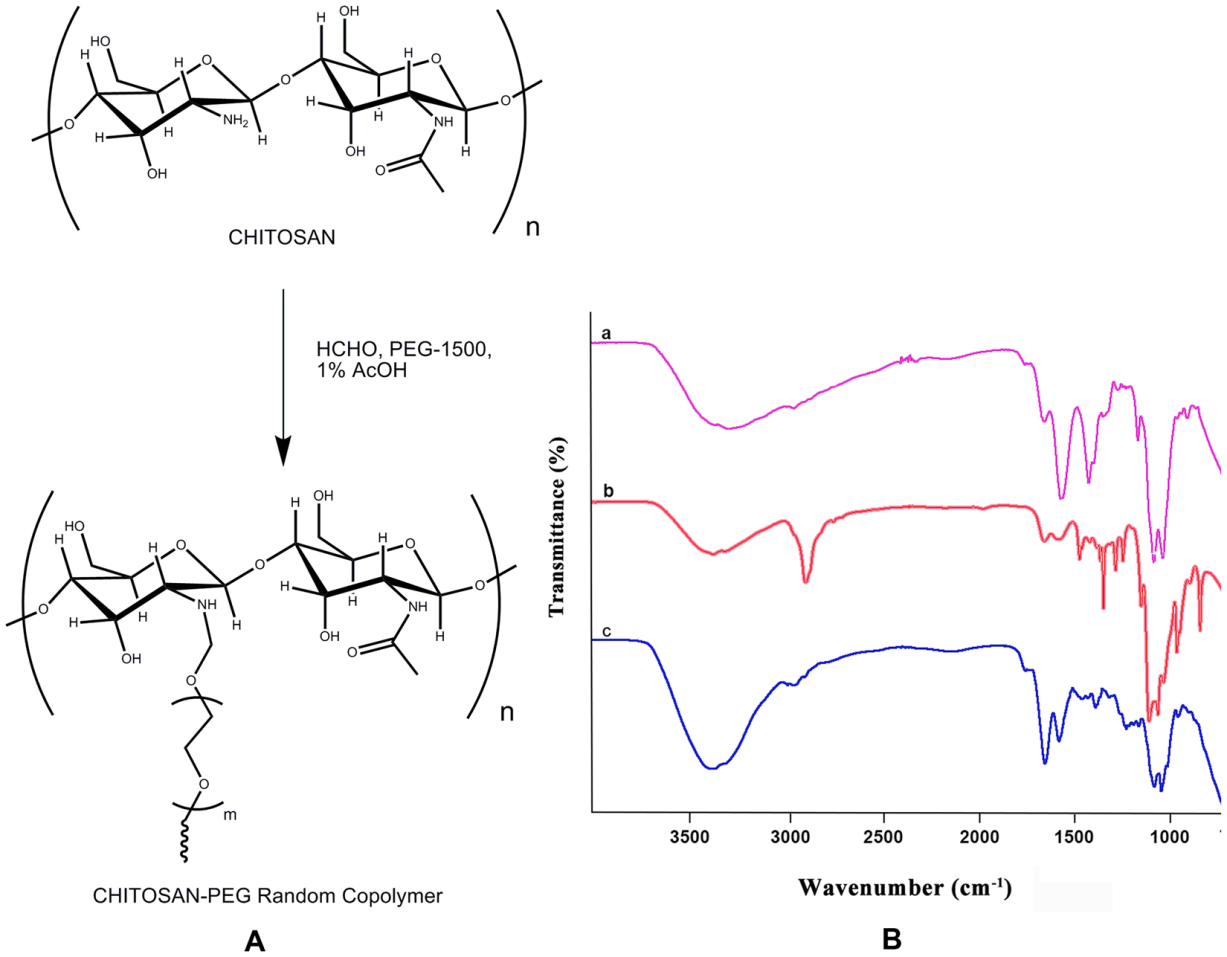
(**A**) Scheme for synthesis of CS-PEG copolymer by formaldehyde linkage method. (**B**) FTIR spectra of (a) Chitosan, (b) CS-PEG and (c) CS-PEG-Hep.

(Fig. 1A) shows the synthesis method of CS-PEG, in which PEG moieties were grafted onto the chitosan molecule by using formaldehyde as a linker. Formaldehyde reacted with the primary amino group of chitosan to produce Schiff-base. Then, the hydroxyl group of polyethylene glycol reacted with this Schiff-base to form CS-PEG. The IR spectrum of chitosan exhibited characteristic bands of 1664 (amide I), 1548 (amide II) and 1407 cm^−1^ (amide III) and its saccharine structure was characterized by the absorption bands at 1152 cm^−1^ (asymmetric stretching of C–O–C bridge), 1070 (C–O stretching). N–H and O–H stretching vibrations were characterized by the broad band in the region of 3200–3500 cm^−1^. For the CS-PEG sample, the peaks corresponding to the hydroxyl group, amino group and amide group of chitosan shifted slightly, and their intensities were significantly reduced as a result of PEG grafting. Compared to the amide I peak at 1664 cm^−1^, the peak intensity of amide II significantly decreased. This resultant spectrum shows that the –NH2 groups of chitosan were partially grafted with PEG. If the chitosan were fully grafted, the peaks corresponding to –NH2 groups at 1571 cm^−1^ would disappear and form a single peak after completion of the reaction. The characteristic peaks associated with PEG in CS-PEG at 1280, 947, and 843 cm^−1^ were significantly decreased. The peaks at 1147 and 2884 cm^−1^ in CS-PEG were attributable to the superposition of C–O, and C–H stretching vibrations of chitosan and PEG. The characteristic absorption band (S O asymmetric stretch) at 1217 cm^−1^ for the associated sulfate groups (2-O, 6-O-sulfation and N-sulfation) were attributed to the conjugated heparin in CS-PEG-Heparin (Fig. 1B).

### NMR spectra and DSC analysis

**Figure 2:**
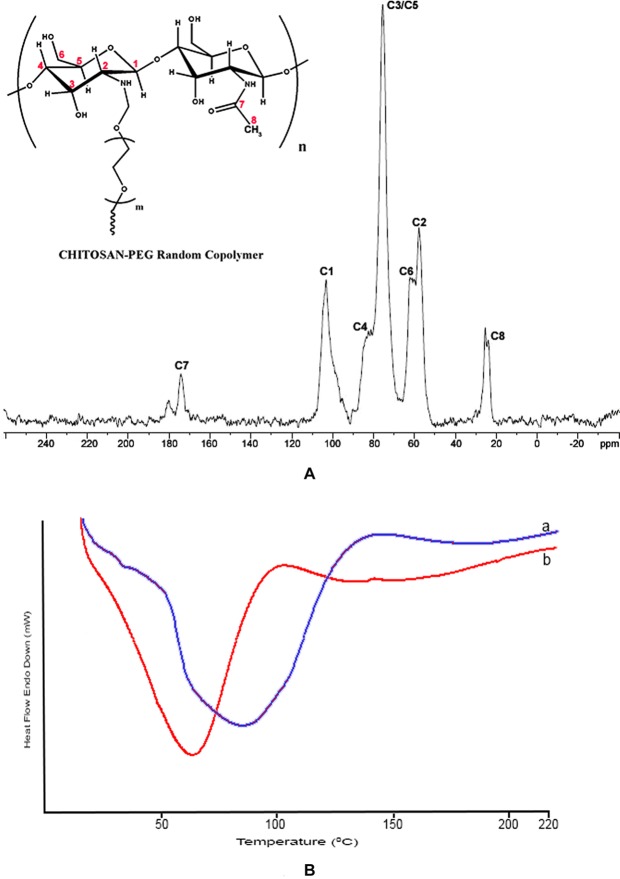
(**A**) 13C solid state NMR spectra of CS-PEG scaffold. **(B)** DSC analysis - comparison of thermograms of (a) Chitosan (b) CS-PEG.

The ^13^C NMR spectra showed peaks corresponding to all the carbon present in chitosan. Peaks at 103.192 correspond to C1, 25.359 to C2, 75.521 to C3 and C5, 82.491 to C4, 174.209 to C7 and 23.874 ppm to C8 were attributed to the polysaccharide structure. The other peaks present are at 60.2, 62.119, 70.8 and 180.148 ppm which corresponds to PEG (Fig. 2A). Thermal properties of the hydrogels were examined by DSC method. The endothermic peak at 80 °C represents the energy required to vaporize water present in the film and was attributed to water loss. The endothermic peak at 60 °C was associated with loss of water from the CS-PEG scaffolds. The changes in the peak can be attributed to the successful grafting of PEG to chitosan (Fig. 2B).

### Swelling and degradation study

**Figure 3:**
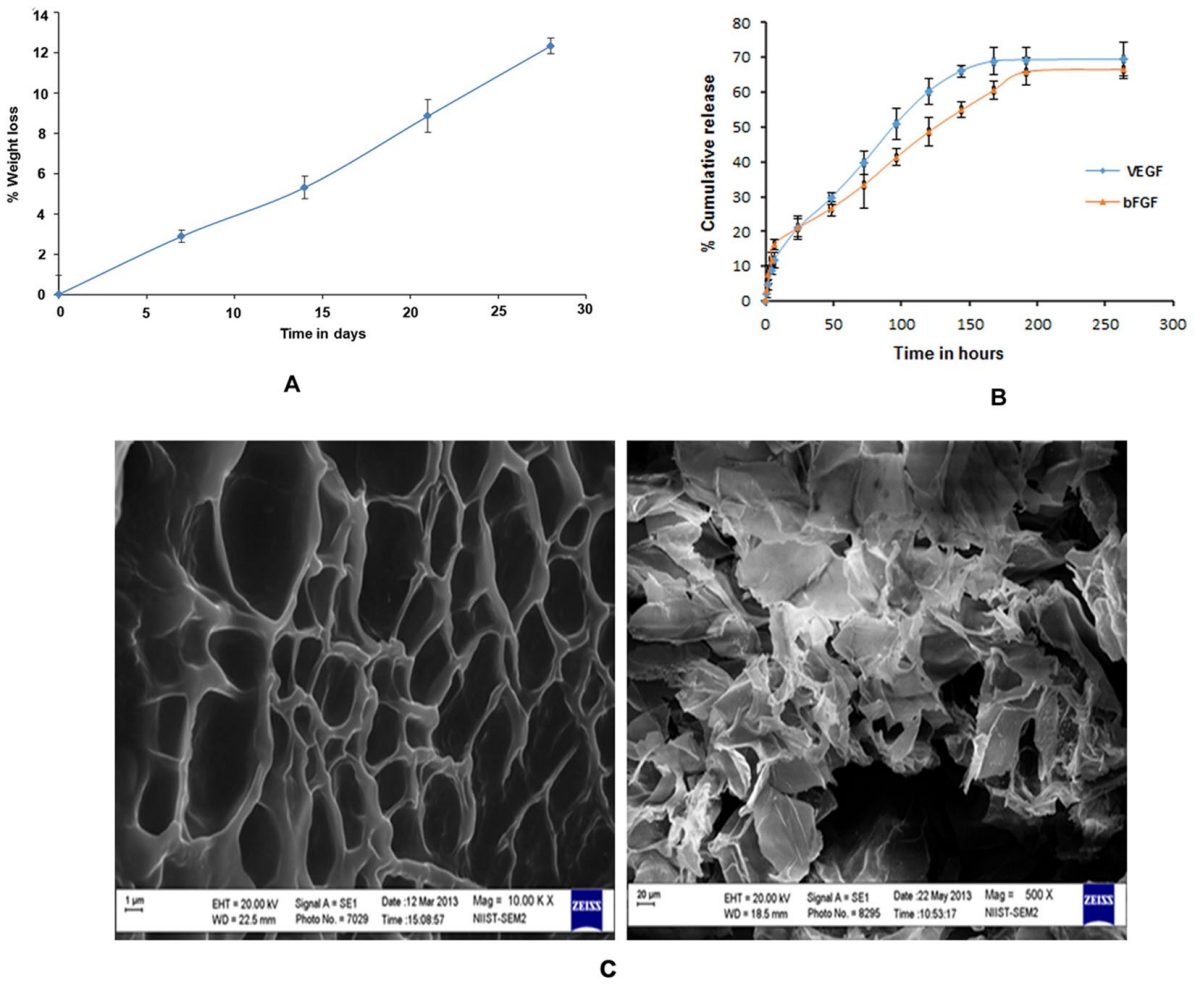
(**A**) Percentage weight loss of CS-PEG scaffold in 1 mg/ml lysozyme in PBS at 37 °C. n = 5. **(B)** Cumulative percentage *in vitro* release profile of bFGF and VEGF from CS-PEG scaffold in PBS (pH 7.4) at 37 °C. (**C**) SEM images of (a) CS-PEG scaffolds, (b) growth factor bound CS -PEG scaffolds. (**C**), reproduced in RGCB annual report.

The equilibrium swelling of CS scaffolds and CSPEG scaffolds were determined to be 280% and 300% respectively. The presence of crosslinker did not show a significant effect on the water absorption (p > 0.05) which may be due to the crosslinking of chitosan by PEG that reduces the free amino groups in chitosan. Chitosan is mainly degraded by lysozyme in the human body. It has been reported that the degradation rate increases with the PEG content which may be due to the crosslinking of chitosan by PEG which may break the intra and inter-molecular hydrogen bonds and may facilitate the access of lysozyme to the binding site. The degradation of CSPEG in the 30 day period was found to be 12% of their weight which is consistent with earlier reports^30^ (Fig. 3A).

### Heparin conjugation and growth factor binding analysis

Heparin conjugation was done to increase the binding efficiency of the growth factors to the scaffold. The amount of heparin on the surface of CS-PEG scaffolds was further quantitatively determined by the toluidine blue colorimetric method. The amount of heparin bound to the scaffold was found to be 309.85 µg per mg of the scaffold. The growth factors bFGF and VEGF were bound to the scaffold, and its binding efficiency and release kinetics were studied by ELISA. bFGF and VEGF showed high binding efficiency of 83% and 86% respectively.

### Surface morphology analysis and growth factor release study

SEM observation of the CS-PEG scaffolds showed a continuous structure of irregular interconnected pores. On addition of growth factors, the surface morphology changed and resulted in a rough surface (Fig. 3C). CS-PEG-Hep scaffold showed a continuous bFGF and VEGF release pattern over two weeks after a moderate burst release. The release profile of the growth factors from the scaffold could be attributed to the strong electrostatic interaction between growth factors and heparin. Growth factor heparin complexes formed strong electrostatic interaction resulting in the slow release of bFGF and VEGF from the scaffold (Fig. 3B).

### Biological evaluation of scaffold

**Figure 4:**
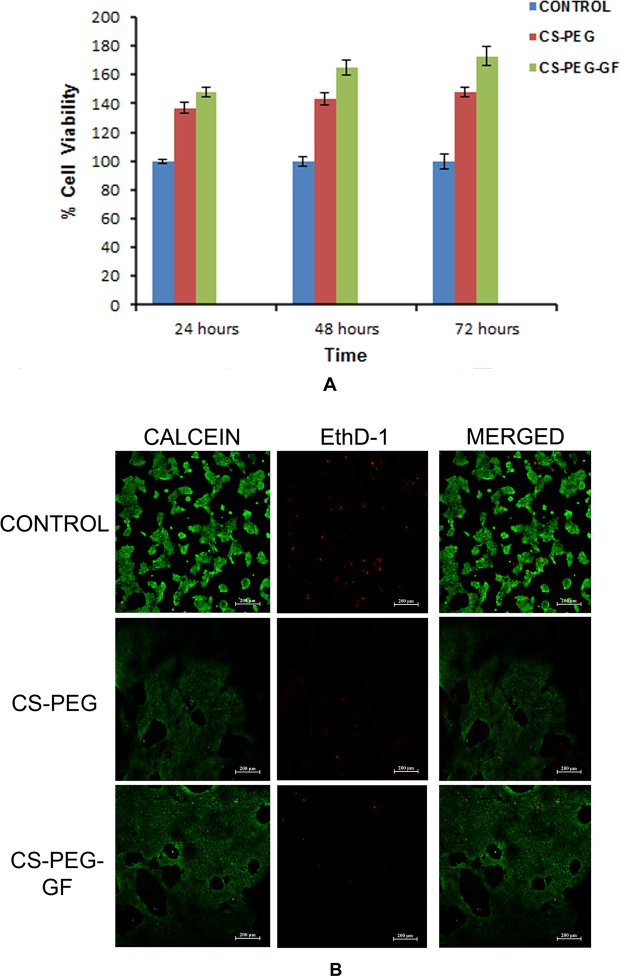
(**A**) Cell proliferation on scaffolds using HaCaT cells. 1. Free tissue culture plate served as control. 2. Plain CS-PEG scaffold and 3. CS-PEG scaffold with Growth Factors. **(B)** Live/dead cell assay of HaCaT cells on control, plain CS-PEG scaffold and CS-PEG scaffold containing growth factors three days after cell seeding, (**B**) reproduced in RGCB annual report.

To investigate whether CS-PEG heparin scaffold can support HaCaT proliferation and survival, MTT and live/dead assays were performed after three days of culture of HaCaT cells on different substrates. It was found that the presence of growth factors enhanced the proliferation and survival of HaCaT cells (Fig. 4A,B). SEM imaging of HaCaT cell-seeded scaffold after three days of culture was done and the morphology of the cells on the scaffold was observed. In confocal image, the cells migrated throughout the scaffold and proliferated confirming the biocompatibility of the scaffold (Fig. S5).

Rate of re-epithelialization was determined using wound healing rate and the area calculated by the Image J software. The rate of healing was faster and more consistent in animals that received, CS-PEG scaffold and growth factor incorporated scaffold treatments on day 10. From day 15, most wounds from all 3 groups were completely healed. Periodical observation of the wounds during the healing process determined that the greatest amount of wound contraction was in the growth factor-treated animals on day 10 and the rate of wound contraction in the control animals was much lower compared to the treated animals (Fig. S2).

**Figure 5:**
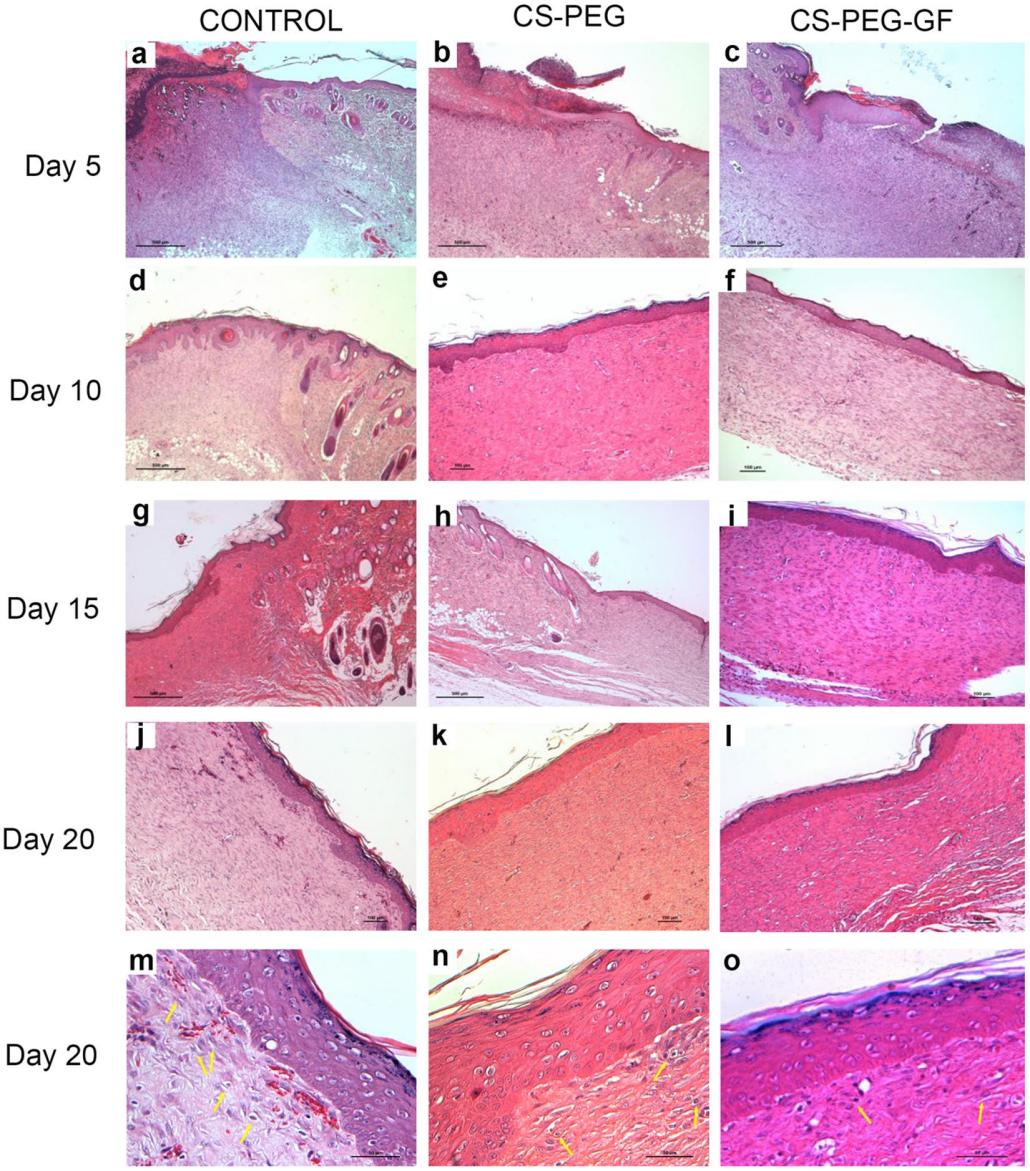
Histological evaluation of full thickness wounds treated with control (**a**,**d**,**g**,**j**), CS- PEG (**b**,**e**,**h**,**k**) and CS-PEG-H scaffold containing growth factors (**c**,**f**,**i**,**l**) for 5,10,15 and 20^th^ days post wounding. (**m**–**o**) Shows the inflammatory cells under higher magnification. (Arrows indicate inflammatory cells).

On day five post-wounding, nonviable necrotic tissues and inflammatory cells were found in the wound area of all groups. Growth factor containing CS-PEG-H scaffold treated wounds showed the presence of a lower number of inflammatory cells and an increased amount of fibroblast-like cells. The epidermis was also noted at the edge of the wound area. Fifteen days post wounding; there was a complete formation of stratified epithelial layer in all groups. Quantitative analysis of blood vessels in the histological sections showed no significant difference between all groups during the later period of wound healing (Fig. S3). But on 10^th^ day, there was significant increase in blood vessels in scaffold treated groups compared to the control. On 15^th^ day of post wounding, we could find the presence of skin appendages in both CS-PEG scaffold group as well as growth factor containing CS-PEG-H group. However, they were not seen in wounds of control group. On day 20 post-wounding, the stratified epithelial layer formed in growth factor containing CS-PEG-H scaffold group was thicker compared to the control and CS-PEG scaffold group (Fig. 5).

**Figure 6:**
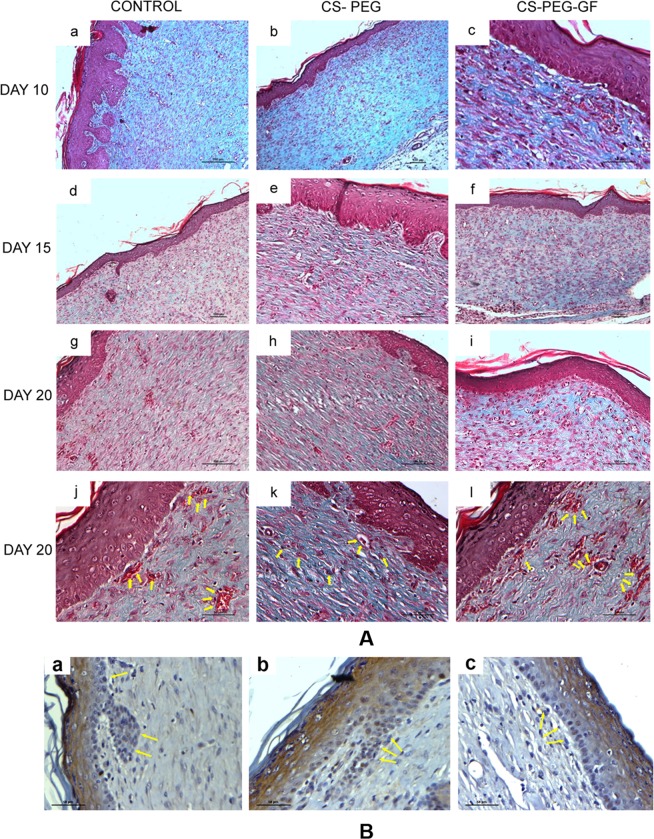
(**A**) Histochemical staining of total collagen with Masson’s Trichrome of wounds treated with control (a,d,g), CS-PEG (b,e,h) and CS-PEG-H scaffold containing growth factors (c,f,i) for 10,15 and 20^th^ days post wounding. (j–l) shows the neovascularized structure under high magnification. (Arrows indicate new capillaries), (**A**) reproduced in RGCB annual report. (**B**) Proliferating Keratinocytes stained using Ki67 immunochemistry in wound sections treated for 20^th^ days with control (a), CS-PEG scaffold (b) and CS-PEG-H scaffold containing growth factors (c). Black arrow shows proliferating keratinocytes.

Masson’s Trichrome staining was done to assess the collagen deposition. The growth factor incorporated CS-PEG-H scaffold treated group showed the presence of higher amount of collagen arranged as aligned fibers compared to the other two groups. There was also an increased amount of myofibroblast formation in wounds treated with growth factor incorporated CS-PEG-H scaffold compared to plain CS-PEG scaffold treated and control wounds. On day 10 and 15 post-wounding, the presence of inflammatory cells was also less in growth factor incorporated CS-PEG-H scaffold treated wounds in comparison with the other groups (Fig. 6A).

Wound sections on day 20 were immunostained with Ki67, a cell proliferative marker to study the effects of growth factor bound CSPEG scaffolds on the keratinocyte behavior. Ki67 positive cells were mainly observed in the control and wounds treated with plain CS-PEG scaffold compared to the wound treated with growth factor containing CS-PEG-H scaffold as seen from morphometric analysis (Figs. 6B and S4).

## Discussion

Chitosan is widely used in wound healing due to its many advantages which include biocompatibility, biodegradability, hemostatic activity, and antibacterial properties^31^. However, chitosan degrades rapidly in an acid environment that is often formed in wounds. Degradation of chitosan *in vivo* depends on various factors like lysozymal degradation, degree of deacetylation and local pH. Acidic conditions have been found to enhance lysozymal hydrolysis (pH = 4.5–5.5). Lysozymal hydrolysis also decreases with increase in degree of deacetylation^32,33^.

Various crosslinking treatments have been used to increase the structural stability of chitosan^34^. PEG is a neutral, water-soluble, non-toxic polymer and has been used in a wide range of biomedical applications^35^. The crystalline structure of chitosan is elucidated by the peak at 80. The intensity of the peak decreased to 60 after crosslinking of chitosan with PEG using formaldehyde as linker due to the reduction in crystalline structure of chitosan. The scaffold was prepared by freeze-drying technique to form a porous three- dimensional biodegradable scaffold^36^. The morphology of scaffolds plays an important role in tissue regeneration as they should have a suitable structure which helps in adhesion, proliferation of cells and exchange of nutrients and oxygen^37^. The pores were visualized using SEM images, and the scaffold was found to have interconnected pores. The surface area of the scaffolds increases as there is an increase in the pore size and total porosity during swelling of the scaffolds. A higher degree of swelling was found to indicate larger surface area/volume ratio thereby paving the way for maximum cell infusion into the scaffold. This also promotes maximum cell growth by attaching to the scaffold surfaces. Though the swelling of scaffolds promotes cell adhesion, it has been found to lower its mechanical properties^38^.

For the controlled release of growth factors, various methods like chemical conjugation of growth factors to the scaffolds have been developed. But this can lead to a potential loss of biological activity of the growth factors after chemical coupling^39^. Hence intermediate molecules like heparin have been used for binding of growth factors. Various growth factors interact with heparin sulfate proteoglycans of the ECM specifically and are known as heparin-binding growth factors like bFGF and VEGF. Hence heparin or heparin sulfate-mimetic molecules have been used to modify the biomaterials to bind heparin-binding growth factors and help in their controlled release. Heparin protects growth factors from proteolytic degradation and promotes their interaction with cell receptors, and hence has been widely used in the delivery of heparin-binding growth factors^39–41^.

Growth factor therapies have the potential to overcome the shortcomings of current wound care problems^42^. Growth factors are critical factors that orchestrate the wound healing process^43^. During wound healing, interactions between ECM and surrounding cell-signaling molecules lead to the expression of growth factors and cytokines which ultimately lead to the formation of new tissues. Finely tuned delivery of growth factors leads to external modulation of cell-signaling events thus altering the wound environment and enabling orderly regeneration^44^.

Fibroblast growth factors stimulate the proliferation and migration of major cell types involved in wound healing like capillary endothelial cells, vascular endothelial cells, fibroblasts, keratinocytes and epithelial cells both *in vitro* and *in vivo*. FGF-2 or bFGF also stimulates epithelialization, neovascularization collagen synthesis, and the formation of granulation tissue in animal models^45^. VEGF is secreted during wound healing by platelets, macrophages, fibroblasts, and keratinocytes and it acts on endothelial cells to promote wound angiogenesis. VEGF increases vascular permeability; degradation of the basement membrane, endothelial migration and proliferation of vascular cells within the wound bed^46^. Providing exogenous growth factors accelerates the process of wound healing but applying them in a sustained and localized fashion is necessary for it to be an effective as growth factor have short half-lives, rapid dilution in the body, and also undesirable effects at high systemic levels^19,47^.

The growth factors exhibited two distinct release mechanisms. One was an initial burst release from CS-PEG-H scaffold which was caused mainly by a quickly dissolving of the surface adsorbed growth factors, and the other was a sustained release controlled by a thermodynamic equilibrium between the growth factor-heparin complexes and free growth factors in release medium. Hence, using CS-PEG-H scaffolds to bind bFGF and VEGF was an effective method for controlling and sustaining growth factor release. This dual delivery system helps in avoiding the need for daily applications and frequent changes in wound dressing, as it allows therapeutic levels of growth factors to be present at the wound bed over time^17,48^.

The *in vitro* proliferation of HaCaT cells induced by bFGF and VEGF released from CS-PEG-GF scaffolds was evaluated to test their biological activity by MTT assay. Both CS-PEG and growth factors incorporated CS-PEG-H scaffolds exhibited cell adhesion activities and were found to promote migration of cells throughout the scaffold. The SEM imaging of HaCaT seeded scaffolds showed the migration and proliferation of cells on the scaffold^49,50^.

CS-PEG-H growth factor scaffold treated group and CS-PEG group showed increased wound healing rate compared to control on day 10 (Fig. S1). There was also increased neovascularization on day 10^th^ post-wounding in growth factor containing scaffold group compared to the other groups. Skin appendages were also present at day 15^th^ in both CS-PEG scaffold group as well as CS-PEG growth factor releasing scaffold, which was absent in the control group. Growth factor containing CS-PEG-H scaffolds showed an increased presence of myofibroblast cells which can synthesize and deposit the ECM components that replace the provisional wound matrix eventually and plays a major role in the contraction and maturation of the granulation tissue, thus remodeling the ECM^51^. 20^th^ days post wounding, the wounds treated with growth factor based CS-PEG-H scaffolds had the lowest collagen content for which the reason might be the increase in mature collagen formation. This indicates that the wound healing has reached the remodeling phase compared to the other groups. These results indicate that growth factor incorporated CS-PEG-H scaffolds have better healing capacity. This was supported by H and E and Masson trichrome staining. Expression of Ki67 in wounds increases with healing up to the proliferation phase and is reduced during re-epithelialization phase. In this work, the immunohistochemical analysis for Ki-67, 20^th^ days post wounding, wounds treated with scaffolds containing growth factors showed the lowest number of proliferative cells indicating that it has entered the next phase of wound healing compared to the other groups. Growth factors based CS-PEG-H scaffolds help in the controlled release of VEGF and bFGF thus promoting the healing process by delivering the growth factors in a controlled and sustained manner. They are a promising candidate for ideal wound healing applications.

## Conclusions

We prepared a heparin-based Chitosan-Polyethylene glycol (CS-PEG-H) scaffold which can deliver dual growth factors VEGF and bFGF to the wound site in a controlled manner. CS-PEG scaffold was synthesized by the freeze-drying method and was characterized by FTIR, NMR, DSC, and SEM. Heparin was added to the scaffold as an intermediate linker, to electrostatically bind the growth factors to the scaffold and helps in preserving the biological activity of the growth factors while protecting them from degradation. The amount of heparin bound was calculated by toluidine blue quantification and the binding efficiency, and release kinetics was calculated by ELISA. The efficiency of the scaffold for wound healing was tested *in vitro* by cell cytotoxicity assay and live/dead cell assay and *in vivo* by full thickness wound model. Histology of the treated wound tissue was evaluated by H and E staining, Masson’s Trichrome staining, immunohistochemical staining of Ki67 antibodies and it was found that the developed system had enhanced wound healing property.

## Material and Methods

Low molecular weight chitosan (448869 from sigma, molecular weight 50,000–190,000 Da, degree of deacetylation >75%), Polyethylene glycol (PEG) (20243.6, sigma average Molecular weight-1,305–1,595), formaldehyde, lysozyme, 1-Ethyl-3-(3-dimethylaminopropyl) carbodiimide (EDC), N-hydroxysuccinimide (NHS), basic fibroblast growth factor (bFGF), vascular endothelial growth factor (VEGF), 3-(4,5-dimethylthiazol-2-yl)-2,5-diphenyltetrazolium bromide (MTT) were purchased from Sigma Aldrich. Acetic acid and Heparin was supplied from Merk. Acetic acid, Heparin, 2-propanol, and xylene was supplied from Merk. VEGF, bFGF and ELISA kits were procured from R and D biosystems. Fetal bovine serum (FBS) was purchased from Pan America. DMEM and Live/dead assay kit was from Invitrogen.

### Synthesis of chitosan-PEG (CS-PEG) scaffold

CS-PEG was prepared by formaldehyde linkage method. 1 g Chitosan was dissolved in 1% acetic acid, and 750 mg of PEG of molecular weight 1500 Da was added along with 30 µl of formaldehyde to it and allowed to react for 24 hours at room temp. The resulting gel was dialyzed against double distilled water for 48 hours, freezed at −20 °C and then lyophilized for 48 hours using OPERON (opr20110824-E01) to form a scaffold.

### Characterization of the scaffold

The copolymer was characterized using FTIR and NMR. Spectra were recorded between 4000 and 600 cm^−1^ wave number range was recorded using Nicolet TM 5700 spectrometer; Thermo Fisher Scientific, Waltham, MA. The ^13^C CP-MAS solid-state NMR measurements were conducted on a Bruker DSX-400 CP- MAS instrument operating at 75.47 MHz. The polymer matrix was analyzed by Differential Scanning Calorimetry (DSC). DSC thermograms were obtained using Pyris TM DSC 6000; PerkinElmer, Waltham, MA. Samples were crimped in standard aluminum pans and heated from 40 °C up to 250 °C at a rate of 10 °C/minute under constant nitrogen purging of 10 ml/minute. An empty pan, sealed similarly to the sample, was used as a reference. The surface morphology of the scaffolds was characterized using scanning electron microscopy. The samples were gold coated and photographed using JEOL, Ltd., JEOL JSM-6490LA.

### Water uptake studies

Swelling studies were performed in PBS at pH 7.4 and temperature of 37 °C to determine the percentage of water absorption. Scaffolds were placed in PBS buffer solution at pH 7.4 and after a predetermined time (24 h) scaffolds were taken out, surface adsorbed water was removed by filter paper. The dry weight of the scaffold (Wo) and wet weight (Ww) was recorded, and the percentage of swelling was determined using the following formula:

$${\rm{Swelling}}\,{\rm{percentage}}=[({\rm{Ww}}-{\rm{Wo}})/{\rm{Wo}}]\times 100$$

### *In vitro* biodegradation studies

The biodegradation rate of the samples was tested by measuring the change in sample weight over time under simulated physiological conditions. The initial weight (dry) of samples was measured (Wi), and the samples were immersed in 90 ml of 0.1 M PBS containing 100 units/ml of lysozyme, and this was maintained at the temperature of 37 °C. The sample was removed from the degradation medium every seven days and rinsed thoroughly with water and lyophilized for 24 h after which the weight was recorded (Wf). Percentage weight remaining was calculated using the formula below:

$${\rm{Weight}}\,{\rm{remaining}}\,( \% )=100-\{({\rm{Wi}}-{\rm{Wf}})/{\rm{Wi}}\}\times 100,$$

where (Wi) is the dry weight of the sample before degradation, (Wf) is the dry weight of the sample after degradation.

### Heparin conjugation to CS- PEG scaffold

Carboxylic acid groups of heparin (H-COOH) were activated using EDC chemistry. To 1 mg/ml solution of heparin in 0.05 M MES buffer (pH 5.0), 25 mM EDC and 10 mM NHS was added. After 10 min the CS-PEG scaffold was added to the activated heparin solution and incubated for 4 hours with gentle agitation at 35 °C. It was washed two times with MES buffer, frozen at −20 °C and was then lyophilized for 24 hours.

### Quantification of heparin conjugated to the scaffold

Heparin conjugated to the surface of CS-PEG scaffold was determined by the toluidine blue colorimetric method. 10 mg scaffold was taken and incubated with 1 ml of TBO reagent for 2 hours with gentle agitation. The samples were then rinsed with distilled water for 5 minutes. The samples were washed twice. The residual dye was then solubilized with a mixture of 0.1 M NaOH and ethanol in the ratio 1:4 and the absorbance were measured at 530 nm.

### Determination of bFGF and VEGF bound to CS-PEG-H scaffold

The CS-PEG-H scaffolds were immersed in 1 ml PBS (pH 7.4) containing 500 ng of bFGF and 500 ng of VEGF. The scaffolds were incubated for 1 h at room temperature on a shaker. The supernatants were collected and the growth factor bound scaffolds were washed with PBS twice. All the washings were also collected, and mixed with previous supernatants. The amount of bFGF and VEGF in the collected solution was assayed using a Quantikine Immunoassay kit according to the manufacture’s instruction (Human bFGF Quantikine ELISA kit and Human VEGF Quantikine ELISA kit, R & D Systems, Minneapolis, MN, USA).

Binding efficiency of bFGF and VEGF to the CS-PEG-H scaffolds was calculated according to the following formula:

$${\rm{Binding}}\,{\rm{efficiency}}\,( \% )=[({\rm{Wa}}-{\rm{Wb}})/{\rm{Wa}}]\times 100$$

where (Wa) and (Wb) is the weight of bFGF and VEGF respectively in PBS solution before and after incubation of the CS-PEG scaffolds.

### Release determination of bound bFGF and VEGF

The CS-PEG-H scaffolds were immersed in 50 ml PBS of pH 7.4 containing 1% bovine serum albumin (BSA) at 37 °C to perform bFGF and VEGF release test. BSA act as stabilizing agent and prevents the degradation of growth factors during the release. At pre-set time intervals 100 µl of the incubation solution was collected and stored at −20 °C. The amount of bFGF and VEGF in the collected release medium was determined by the Quantikine Immunoassay kits according to the manufacture’s instruction. The growth factor bound scaffold was then frozen at −20 °C and lyophilized for 24 hours.

### Cytocompatibility studies

The cytocompatibility of scaffolds was evaluated using MTT assay. The samples were cut into a pre-determined size and placed in a 24 well plate. They were sterilized with 70–75% ethanol solution for 30 min before cell seeding. Samples were then washed with PBS three times and incubated with a medium for 1 h. HaCaT cells were cultured in DMEM medium supplemented with 10% FBS and penicillin (100 units ml^−1^) and streptomycin (100 μg ml^−1^) at 37 °C in a humidified atmosphere with 5% CO_2_. The cells were harvested and seeded on the scaffolds at a density of 5 × 10^4^ cells/ml and incubated at 37 °C. After 24, 48 and 72 h, MTT was added to the wells and further incubated for 4 h MTT, and the absorbance was measured at 570 nm using microplate reader. Triplicates of samples were done from which cell viability was evaluated.

### SEM analysis of cell-seeded scaffold

CS-PEG-GF scaffold was cut into pieces of pre-determined sizes and was sterilized with ethanol. The scaffolds were then washed thoroughly with PBS. HaCaT cells were then seeded on the scaffolds and incubated for three days. The cells were then fixed in glutaraldehyde and dehydrated in a series of ethanol solutions and dried to a critical point, coated with gold and imaged.

### Live/dead cell assay

Live/dead assay using calcein AM and ethidium homodimer fluorescent staining was performed to evaluate cell viability and proliferation in direct contact with the prepared scaffolds. HaCaT seeded on scaffolds were labeled with calcein AM and ethidium bromide to distinguish the population of live cells from the dead-cell population. Scaffold with cells was incubated with the staining solution prepared according to manufacturer’s instructions, for 15 minutes in the dark. Images were captured using a laser confocal microscope (TCS SP2; Leica Microsystems, Wetzlar, Germany).

### Full-thickness wound model

Adult male Wistar rats weighting of 120–150 g were selected for the animal experiment. The dorsal hair of the rats was removed under isoflurane anesthesia. Skin tissue measuring 1 × 1 cm width was removed surgically to create an excision wound, on the back using sterile surgical tools. Animals were divided into three groups: wound treated with CS-PEG scaffold, wound treated with CS-PEG-GF scaffold and a control group treated with PBS. The scaffolds were applied directly to the wound and were bandaged using adhesives. On 5^th^, 10^th^, 15^th^ and 20^th^ day of post wounding, three rats from each group were sacrificed by carbon dioxide inhalation.

### Histology

Rats from all three groups were sacrificed on 5^th^, 10^th^ and 15^th^ day and 20^th^ day post-wounding and tissues from wound site were removed and fixed with 10% formalin, dehydrated through graded alcohol series, cleared in xylene and embedded in paraffin wax. Sections of 5 μm were cut and stained with hematoxylin and eosin (H&E). Masson’s Trichrome method for staining collagen was performed on paraffin sections, as described previously^52^.

### Determination of wound healing rate

The area of the wounds were calculated using the Image J software to analyze the re-epithelialization by the wound healing rate (WHR)^53^

$${\rm{WHR}}=({\rm{Initial}}\,{\rm{area}}-{\rm{Final}}\,{\rm{area}})/{\rm{Initial}}\,{\rm{area}}$$

The initial area corresponds to the day of the surgery (day 0) and the final area corresponds to the day of euthanasia (day 5, 10, 15, or 20).

### Histomorphometrical analysis of angiogenesis by image analysis

Histological sections stained with H&E were visualized in a Nikon Eclipse 55i microscopic system, Japan. Images were collected using 10X, 20X and 40X magnification objectives. Five fields were randomly selected for every test and control samples. All groups were evaluated for angiogenesis quantification. The Cell Counter ImageJ software was used to count the number of blood vessels and the data was reported as the average number of blood vessels of three samples of each sampling period to both groups.

### Immunohistochemistry assay

20 days post wound healing treatments expression level of Ki67 were investigated by immunohistochemical staining for the detection of proliferative cells. The slides were warmed at 60 °C to melt the paraffin wax and then hydrated through a graded ethanol series. The slides were heated up to 95 °C in a modified citrate buffer to enhance antibody binding. The slides were then incubated with the primary antibody (anti Ki67, 1:800, Thermo scientific) overnight at 4 °C. After washing the slides in phosphate buffered saline (PBS) to remove excess primary antibody, the tissue was then incubated with a horse radish peroxide conjugated anti-rat secondary antibody for 2 h at room temperature and protected from light. Excess secondary antibody was removed by PBS and then the slides were incubated for 3 min with a nuclear stain, hematoxylin. The slides were photographed and the images were analyzed by ImageJ software and immunohistochemistry was scored as number of Ki67 positive cells per high powered field (hpf).

### Statistics

All data are expressed as a mean ± standard deviation, and Student’s unpaired t-test was used to compare means between groups. A p < 0.05 was considered statistically significant.

### Abbreviations

VEGF: vascular endothelial growth factor, bFGF: basic fibroblast growth factor, PEG: Polyethylene glycol, EDC: 1-Ethyl-3-(3-dimethylaminopropyl) carbodiimide, NHS: N-hydroxysuccinimide, MTT: 3-(4,5-dimethylthiazol-2-yl)-2,5-diphenyltetrazolium bromide, FBS: Fetal bovine serum, PBS: phosphate buffered saline, MES: 2-(N-morpholino)ethanesulfonic acid, DMEM: Dulbecco’s Modified Eagle’s Medium, ELISA: enzyme-linked immunosorbent assay, DSC: differential scanning calorimetry, SEM: scanning electron microscope, FTIR: Fourier-transform infrared spectroscopy, NMR: Nuclear magnetic resonance, ECM: extra cellular matrix.

### Ethics approval and consent to participate

All animal experiments were approved by the institutional animal ethics committee at Rajiv Gandhi Centre for Biotechnology.

### Experimental approval

All the experimental methods were performed in accordance with relevant guidelines and regulations.

## Supplementary information

Supplementary information
