# Supplementary material for: PEG grafted chitosan scaffold for dual growth factor delivery for enhanced wound healing
Source: Sci Rep. 2019 Dec 16;9:19165. doi: 10.1038/s41598-019-55214-7 (PMC6915706; doi:10.1038/s41598-019-55214-7)
Supplement: Supplementary file 1 — Supplementary information [file 41598_2019_55214_MOESM1_ESM.pdf]

Supplementary Information

PEG grafted chitosan scaffold for dual growth factor delivery for enhanced wound healing

Amritha Vijayan,[1a] Sabareeswaran A,[2] G.S. Vinod Kumar[1 *]

[1]*Cancer Biology, Nano Drug Delivery Systems (NDDS), Bio-Innovation Center (BIC), Rajiv Gandhi Centre for Biotechnology, Thycaud P.O, Thiruvananthapuram, Kerala, India-695014.*
[2]*Histopathology laboratory, Sree Chitra Tirunal Institute for Medical Sciences & Technology, Thiruvananthapuram, Kerala, India-695 011.*
[a] *Research Scholar, Dept. of Biotechnology, Faculty of Applied Science & Technology, University of Kerala, Trivandrum, Kerala, India. 695581.*

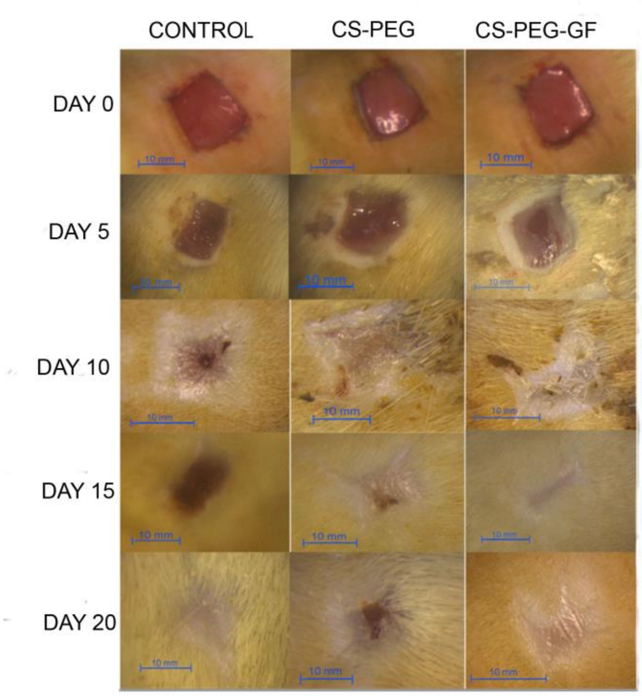

Fig. S1. Photographs of macroscopic appearance of wounds treated with control, CS-PEG and CS-PEG-GF at 5, 10, 15 and 20 days post wounding.

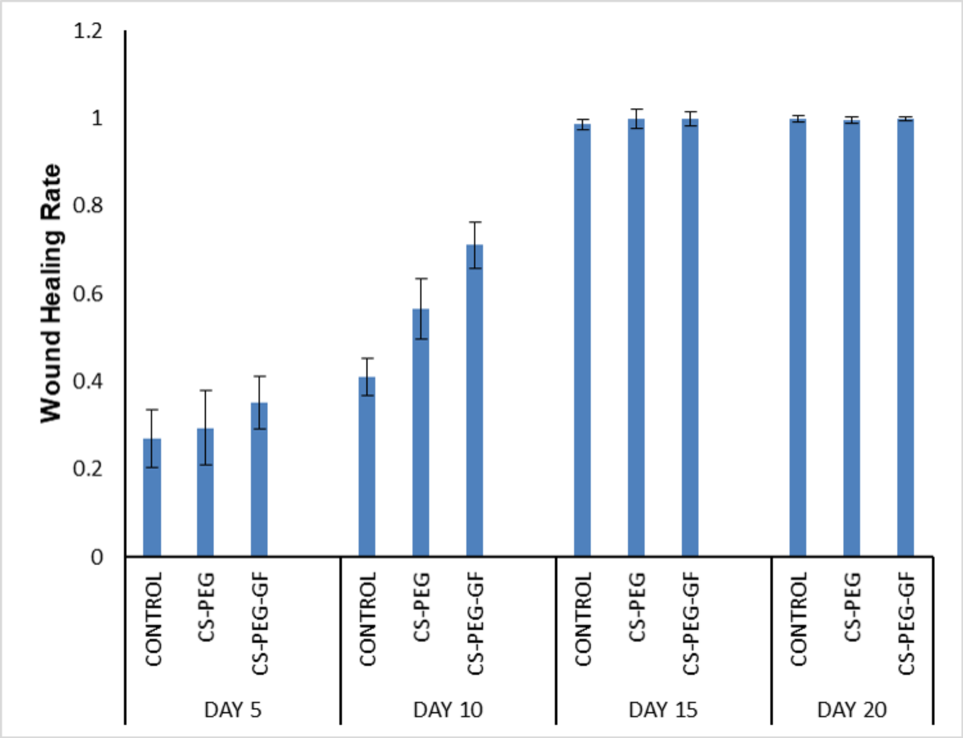

Fig. S2. Evaluation of treated wounds (a) re-epithelialization of wounds treated with control, CS-PEG and CS-PEG-GF at 5, 10, 15 and 20 days post wounding.

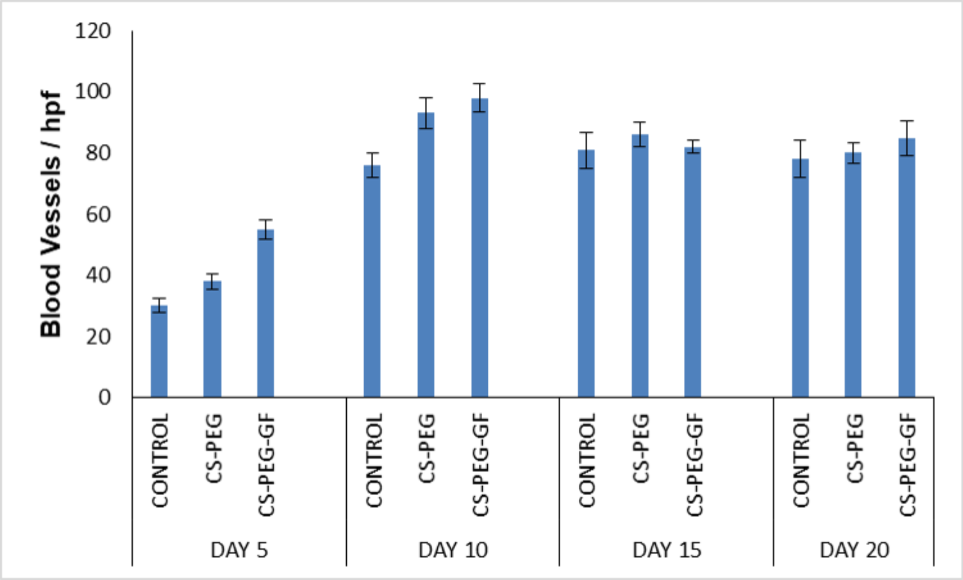

Fig. S3. Blood vessels histomorphometry of wounds treated with control, CS-PEG and CS-PEG-GF at 5, 10, 15 and 20 days post wounding.

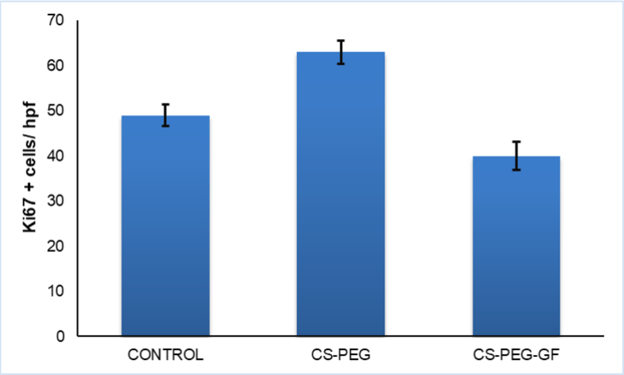

Fig. S4. Quantitative analysis of KI67 positive cells in wounds treated with control, CS-PEG and CS-PEG-GF at 20 days post wounding.

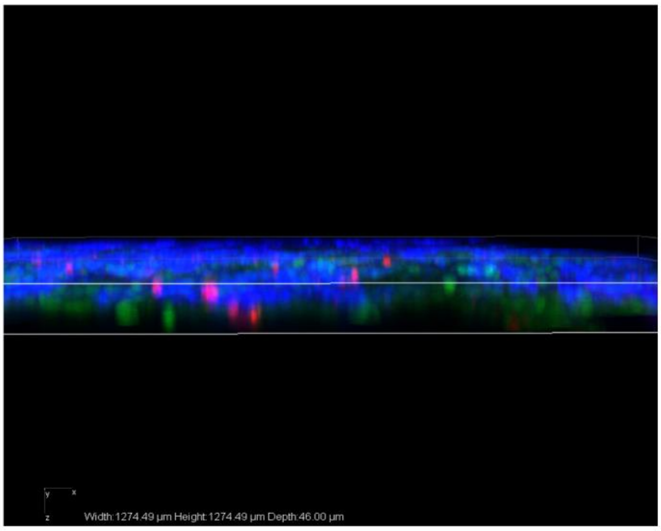

A

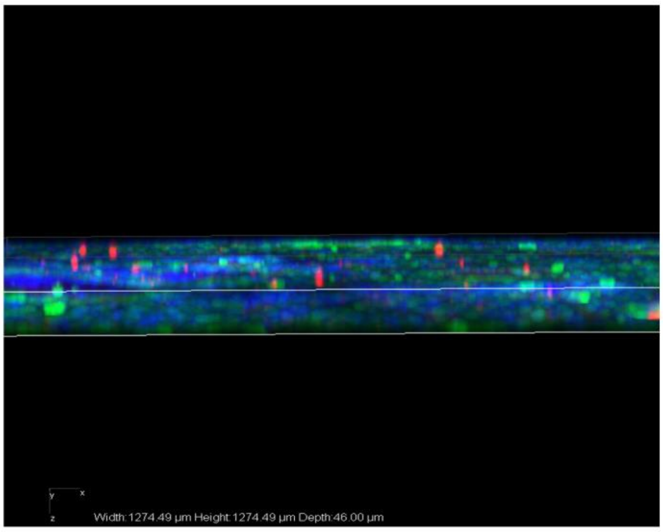

B

Fig. S5. Confocal images of cells grown on (A) CS-PEG and (B) CS-PEG-GF. The live cells were stained green by Calcein and dead cell stained red due to EthD-1. The blue channel shows autofluorescence of Collagen- PEG scaffold.
